# Cellulose Crystallinity in Orange Biomass-Derived Cellulose: Comparative Assessment by FT-IR, Solid-State ^13^C NMR, and X-Ray Diffraction

**DOI:** 10.3390/molecules31213833

**Published:** 2026-10-23

**Authors:** Symone Costa de Castro, Matheus Signorelli Corrêa, Ljubica Tasic

**Affiliations:** 1 Laboratory of Biological Chemistry (LQB), Institute of Chemistry, Universidade Estadual de Campinas (UNICAMP), Campinas 13083-970, SP, Brazil; s232970@dac.unicamp.br (S.C.d.C.); m236586@dac.unicamp.br (M.S.C.); 2 Instituto Federal de Educação, Ciência e Tecnologia Farroupilha (IFFar), Alegrete 97555-000, RS, Brazil

**Keywords:** citrus biomass, cellulose crystallinity, FT-IR, solid-state ^13^C NMR, X-ray diffraction, pectin, biomass valorization

## Abstract

Reliable determination of cellulose crystallinity is essential for biomass valorization, yet commonly used analytical methods may respond differently to residual non-cellulosic components present in partially purified lignocellulosic materials. In this study, fourteen cellulose-rich samples were produced from depectinated orange biomass under controlled alkaline processing conditions and characterized by Fourier-transform infrared spectroscopy (FT-IR), solid-state ^13^C nuclear magnetic resonance (CP/MAS ^13^C NMR), and X-ray diffraction (XRD). Crystallinity indices (CIs) were calculated using the lateral order index (FT-IR), C4 peak deconvolution (ssNMR), and the Segal method (XRD), while principal component analysis was employed to support multivariate interpretation of the FT-IR and NMR datasets. FT-IR-derived CIs ranged from approximately 65 to 91%, with the starting biomass exhibiting the highest value despite its substantial residual pectin content. In contrast, ssNMR-derived CIs were consistently lower (approximately 27–49%) and showed limited sensitivity to residual pectin, indicating greater selectivity toward cellulose crystalline organization. XRD produced intermediate CI values and displayed a moderate positive correlation with ssNMR (r = 0.5) but a moderate negative correlation with FT-IR (r = −0.5). PCA further revealed that pectin-associated FT-IR bands (~1737 and ~1635 cm^−1^) were the primary drivers separating the starting biomass from the purified cellulose samples. The study establishes a practical framework for selecting crystallinity evaluation methods during the sustainable valorization of citrus biomass into cellulose-based materials.

## 1. Introduction

Citrus biomass (CB), a major residue of juice production, is an abundant source of pectin and cellulose. Pectin, largely composed of D-galacturonic acid units forming the homogalacturonan (HG) backbone, is the primary target for CB valorization, especially in regions with strong citrus industries such as Brazil, Mexico, and Europe [1,2,3,4]. After pectin extraction, the resulting solids contain significant amounts of cellulose whose yield varies with citrus type and extraction method [5,6,7]. Therefore, the characterization and valorization of the cellulose-rich fraction obtained after pectin extraction represent an important opportunity for increasing the value of citrus-processing residues.

Cellulose is a linear β-1,4-linked D-glucan whose chains assemble into microfibrils composed of crystalline (ordered) and amorphous (disordered) regions. Several allomorphs of cellulose have been described, with Iα and Iβ occurring naturally and others formed through chemical treatments, like cellulose II [8]. The crystallinity index (CI) of cellulose, defined as the relative proportion of crystalline to amorphous regions, plays a key role in determining thermal stability, reactivity, water sorption, and enzymatic digestibility [9,10,11,12,13]. Thus, CI is a key property of cellulose and precise CI assessment is essential for applications ranging from biofuels to nanocellulose and biodegradable materials [14,15].

The structural characteristics of cellulose-rich fractions can be strongly influenced by the conditions employed during biomass fractionation. Alkaline treatment is widely used for lignocellulosic biomass, including CB, because it can promote the removal or modification of non-cellulosic components and alter the physical structure of the resulting material. In addition to changes in chemical composition, alkaline treatment may affect crystallinity, porosity, surface morphology, fibrillar organization, and chain orientation [9]. The CI of lignocellulosic biomass may increase following pretreatment, particularly when amorphous components such as hemicellulose and lignin are preferentially removed, since cellulose is the principal crystalline component of lignocellulosic biomass [16,17]. However, changes in CI are not necessarily uniform, as they depend on biomass composition, treatment type, and processing conditions. Moreover, treatment-induced modifications in morphology and structural characteristics may affect mass and heat transfer during processing, further highlighting the need to evaluate the response of each biomass under applied conditions [18].

Several analytical techniques have been used to evaluate cellulose crystallinity, among which X-ray diffraction (XRD) is one of the most widely used. XRD provides information on the crystalline and amorphous organization of cellulose and allows CI to be estimated using different approaches, including peak-height methods such as the Segal method, peak-area analysis, peak deconvolution, and amorphous-background subtraction [19,20,21]. The Segal method is particularly convenient because of its simplicity; however, it has recognized limitations associated with the contribution of amorphous regions, peak overlap, and baseline assumptions [8,19,21]. Consequently, different XRD-based approaches may provide distinct CI values even for the same sample.

Solid-state ^13^C nuclear magnetic resonance provides an alternative approach for evaluating cellulose structural organization. In the C4 region, crystalline and amorphous cellulose environments generate distinguishable signals, typically centered at around 89 and 84 ppm, respectively. Deconvolution and integration of these peaks can therefore be used to estimate cellulose crystallinity [22]. Other cellulose carbon environments, including C1, C2/C3/C5, and C6, can provide complementary information on molecular organization, with peaks typically occurring at 102–108, 70–80, and 62–65 ppm, respectively [23,24]. For citrus-derived cellulose, XRD-based CI values have frequently been reported to be higher than those obtained by solid-state ^13^C-NMR [6,7,25,26], highlighting the influence of the analytical approach on the resulting crystallinity values.

FT-IR spectroscopy is widely used to monitor the structural changes occurring during the fractionation of lignocellulosic biomass, particularly after the removal of non-cellulosic components such as lignin and hemicelluloses. Beyond its qualitative application, FT-IR spectra can provide semi-quantitative information on the supramolecular organization of cellulose through indices derived from characteristic vibrational bands. Among the most frequently used parameters are the lateral order index (LOI), calculated from the ratio of the absorbances at 1418 and 894 cm^−1^ (A_1418_/A_894_), the total crystallinity index (TCI), obtained from the ratio between the bands near 1370 and 2900 cm^−1^ (A_1370_/A_2900_), and the hydrogen-bond intensity (HBI), calculated from the ratio of the absorbances around 3336 and 1336 cm^−1^ (A_3336_/A_1336_). These FT-IR-derived ratios have been used to assess cellulose molecular ordering, crystallinity-related changes, and hydrogen-bonding interactions in different cellulose substrates and lignocellulosic materials [27,28,29].

Recent studies have further demonstrated their applicability to cellulose obtained from agricultural and fruit-processing residues. For example, the LOI, TCI, and HBI were employed to evaluate cellulose nanofibers obtained from passion fruit peel, providing semi-quantitative information on molecular order, crystallinity, and hydrogen bonding [29]. Similarly, sequential alkaline–acid–peroxide fractionation of sugarcane bagasse resulted in substantial changes in these FT-IR-derived indices, together with an XRD crystallinity index of 73% for the optimized cellulose fraction, demonstrating the potential of combining FT-IR and XRD to monitor structural changes during biomass fractionation [27]. Despite the increasing application of these parameters to agricultural residues, their use for characterizing cellulose-rich fractions derived specifically from CB remains comparatively unexplored. Their application to citrus-derived cellulose may therefore provide complementary evidence of the structural changes associated with the removal of pectin, hemicelluloses, lignin, and other non-cellulosic constituents during fractionation.

In this study, cellulose-rich fractions were obtained from depectinated orange biomass under fourteen controlled sets of alkaline processing conditions to investigate the effects of processing parameters on cellulose crystallinity and structural organization. Crystallinity-related characteristics were evaluated using FT-IR spectroscopy based on LOI, solid-state ^13^C-NMR based on C4 peak deconvolution, and XRD based on Segal method. The relationships among the parameters obtained from the different techniques were further investigated using correlation and chemometric analyses. The novelty of this study lies in the systematic evaluation of cellulose-rich fractions derived from pectin-extracted citrus biomass and in the assessment of how alkaline conditions affect their structural organization and crystallinity. In addition, the potential of FT-IR and solid-state ^13^C-NMR as complementary approaches to XRD for assessing crystallinity in citrus-derived cellulose was evaluated.

## 2. Results and Discussion

### 2.1. Characterization of the Starting Biomass

The starting depectinated orange biomass, provided by a pectin factory, was characterized in terms of total solids, ash, cellulose, lignin, and pectin content. The measured values were 18.02 ± 0.62%, 0.77 ± 0.31%, 36.47 ± 0.50%, 7.53 ± 0.50%, and 8.62 ± 0.20%, respectively. As expected, the pectin content was lower compared to raw orange bagasse, in which pectin can reach up to 30% of dry weight [4]. Cellulose is the second most abundant biopolymer in citrus biomass; consequently, its relative content increases after pectin extraction. Cellulose and lignin were quantified using the filter bag technique (ADF and NDF). Hemicellulose content is typically estimated by subtracting ADF from NDF; however, in samples with very high cellulose content, this calculation becomes unreliable. When ADF dominates, the subtraction involves two large, similar values, making the result highly sensitive to small analytical errors, such as incomplete detergent action, bag clogging, or over-retention of solubilized components. Additionally, filter bags may retain non-fiber contaminants (e.g., protein, ash), which biases the measurement toward the insoluble fibrous fraction. As a result, reliable quantification of hemicellulose in this depectinated biomass was not feasible using the employed method.

### 2.2. FT-IR Analysis

**Figure 1:**
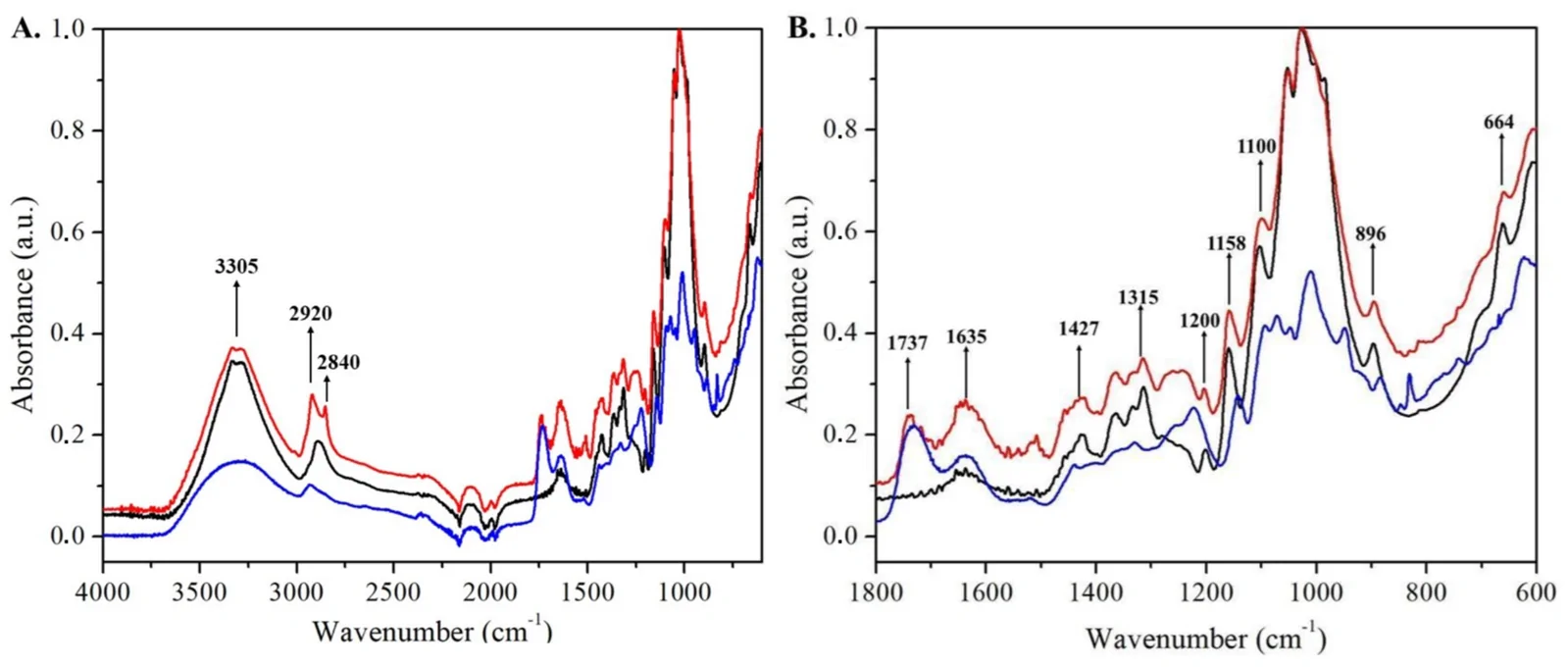
FT-IR spectra of AM0 (red), α-cellulose (black), and pectin (blue). (**A**) Spectra in the range 4000–600 cm^−1^ and (**B**) spectra in the range 1800–600 cm^−1^.

The FT-IR spectra of the starting biomass (AM0), commercial α-cellulose, and pectin extracted from orange peels are shown in Figure 1.

**Figure 2:**
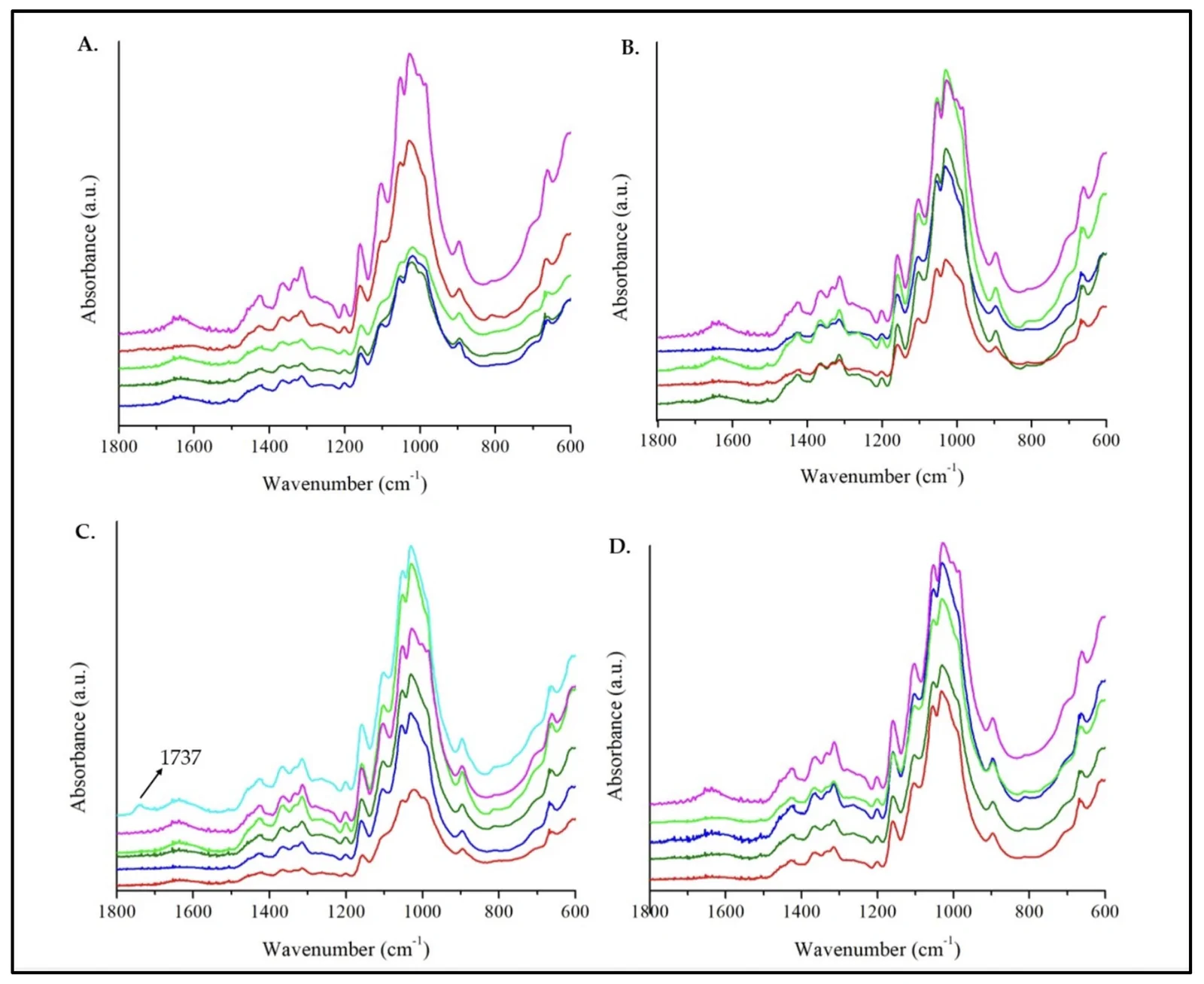
FT-IR spectra of the cellulose samples at different biomass processing in the range of 1800–600 cm^−1^. (**A**) Temperatures: AM1—red, AM2—light green, AM3—dark green, AM4—blue, and alpha-cellulose—purple; (**B**) NaOH concentrations: AM2T (NaOH 3%)—blue, AMC2 (NaOH 5%)—dark green, AMC3 (NaOH 7%)—light green, AMC4 (NaOH 9%)—red, and alpha-cellulose—purple; (**C**) times: AM3—red, AM2.5T—light green, AM3T—dark green, AM4T—light blue, AM2T—dark blue, and alpha-cellulose—purple; (**D**) S/L ratios: AM2T (1/10, red), AMP2 (1/15, blue), AMP3 (1/20, light green), AMP4 (1/25, dark green), and alpha-cellulose (purple).

The spectra revealed characteristic vibrational features of these polysaccharides, particularly within the fingerprint region (1800–600 cm^−1^). A broad absorption band centered around 3305 cm^−1^ was attributed to O–H stretching vibrations, reflecting the extensive hydrogen bonding typical of carbohydrate structures. The peak near 2920 cm^−1^ and 2840 cm^−1^ corresponded to aliphatic C–H stretching [30], present in all samples but slightly more intense in the pectin-rich sample due to contributions from pectin, lignin, and hemicellulose. The ester carbonyl stretching at 1737 cm^−1^, clearly observed in both AM0 and pectin but absent in cellulose, confirmed the presence of esterified galacturonic acid units in the pectin backbone [4,25]. This band is also referred to as C=O stretching vibration of acetyl groups and carbonyl in the xylan and to chemical groups present in lignin, as reported by some authors [18,31]. However, lignin is a minor component in citrus biomass, about 3–5% on a dry basis, while pectin is the major component, up to 25% [32]. Thus in a FT-IR spectrum of an orange and/or citrus biomass in general, the band at 1737 cm^−1^ (or 1740 cm^−1^) is prior referred to ester carbonyl stretching of esterified galacturonic acid [33]. The band at 1635 cm^−1^ was assigned to asymmetric –COO^−^ stretching or adsorbed water bending vibrations, whereas the absorption at 1427 cm^−1^ reflected symmetric carboxylate stretching [9], observed in both pectin and cellulose spectra. Strong bands in the 1200–1000 cm^−1^ region corresponded to C–O–C and C–O stretching vibrations of glycosidic bonds, with the signal at 896 cm^−1^ serving as a marker for β-(1→4)-glycosidic bonds in cellulose [34]. Overall, the AM0 spectrum combined ester/carboxyl bands from pectin with glycosidic fingerprints from cellulose, confirming its hybrid polysaccharide composition. A detailed comparison of the fingerprint regions for all samples and α-cellulose is provided in Figure 2A–D. The intensity of bands at ~1424 cm^−1^ and 896 cm^−1^ varies across samples, reflecting progressive structural rearrangements under different alkaline processing conditions. The 1737 cm^−1^ band, associated with pectic residues, is still detectable in AM4T (Figure 2) but at a much lower intensity than in AM0, highlighting the partial removal of non-cellulosic components while preserving the cellulose framework. These spectral changes provide insights into how processing conditions modulate cellulose crystallinity and chemical composition.

**Figure 3:**
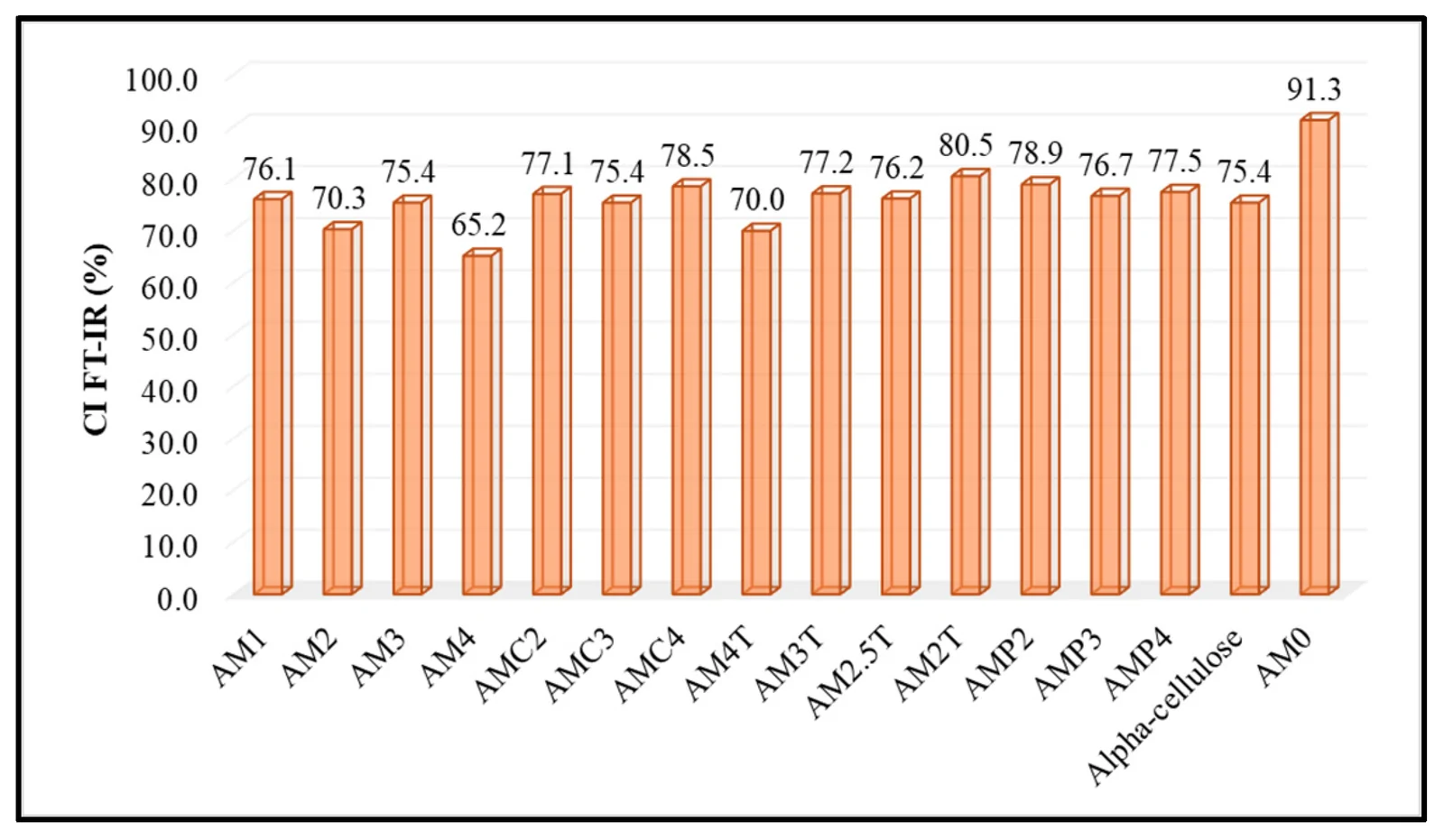
Crystallinity indices (CIs) of the obtained cellulose-based samples. CI was calculated using the lateral order index (LOI), defined as the absorbance ratio at 1424 cm^−1^ and 896 cm^−1^.

Despite the structural information that can be obtained from the FT-IR spectra of cellulose-based materials, the absorbance ratio A_1424_/A_896_, referred to as the crystallinity index (CrI) by Huamani-Palomino et al. [22] and as the lateral order index (LOI) in other studies, was used to assess changes in the structural order of the cellulose samples. As shown in Figure 3, the LOI (expressed as %) values of the cellulose-based samples ranged from approximately 65 to 91%. The untreated starting biomass (AM0) exhibited the highest value (91%), exceeding that of all treated samples and commercial α-cellulose. This unexpectedly high value may be associated, at least in part, with the presence of residual pectin and other non-cellulosic components in the starting biomass. In particular, the contribution of pectin to the FT-IR region around 1424 cm^−1^ may influence the intensity ratio used for the LOI calculation, potentially resulting in an overestimation of the index without necessarily reflecting a higher degree of cellulose crystallinity. A similar trend of FT-IR-based crystallinity-related indices has been reported by Huamani-Palomino et al. [22], who obtained an unusually high value of 250% for a cellulose-rich sample containing hemicellulose and other impurities. The authors associated this result with incomplete bleaching. Although such values should not be interpreted as actual crystallinity percentages, they illustrate the sensitivity of spectroscopic indices to the chemical composition and purity of lignocellulosic samples. Therefore, the relatively high CI observed for AM0 may reflect, at least partially, the contribution of residual non-cellulosic components rather than a greater degree of crystalline ordering of cellulose itself.

The lower CI values observed after treatment may be related to changes in the composition and supramolecular organization of the cellulose-rich fraction resulting from alkaline processing [35]. Alkaline treatment promotes the removal of non-cellulosic components, particularly hemicelluloses and pectic substances, thereby altering the organization and accessibility of the cellulose microfibrils. The extent of these effects depends strongly on the alkali concentration and treatment conditions. Under sufficiently severe alkaline conditions, NaOH treatment can also induce polymorphic transformations of cellulose I into cellulose II, which are accompanied by changes in molecular packing and hydrogen-bonding arrangements [36]. Borysiak and Doczekalska [36], for example, reported that the extent of cellulose I-to-cellulose II transformation in pine wood increased with increasing NaOH concentration and treatment time and was accompanied by changes in the crystallinity of the material.

Interestingly, a similar trend was reported for untreated sugarcane biomass by Terefe et al. [27], which exhibited a higher LOI than the corresponding treated samples. However, these authors interpreted lower LOI values as indicative of a more organized cellulose I lattice. This interpretation highlights the importance of considering LOI as a spectroscopic parameter that is sensitive to cellulose molecular order and sample composition, rather than as a direct and absolute measure of crystallinity. Thus, the decrease in LOI observed for the treated samples in the present study should not be interpreted solely as a reduction in cellulose crystallinity. Instead, it may reflect a combination of changes in cellulose organization and the removal of non-cellulosic components that contributed to the FT-IR bands used in the calculation. The LOI values of the treated samples, which were comparable to that of commercial α-cellulose, are consistent with a greater removal of residual pectin and other non-cellulosic components from the starting biomass.

Among the samples treated with different NaOH concentrations, the highest FT-IR-derived CrI value was observed for AMC4 (9% NaOH, *m*/*m*). In the temperature series, the highest CrI value was obtained for AM1, whereas in the treatment-time series, AM2T (150 min) exhibited the highest value. Variations in the solid-to-liquid ratio had a comparatively limited effect on the FT-IR-derived CrI, with values remaining within a relatively narrow range of 77.4–80.5% for AM2T, AMP2, AMP3, and AMP4.

**Figure 4:**
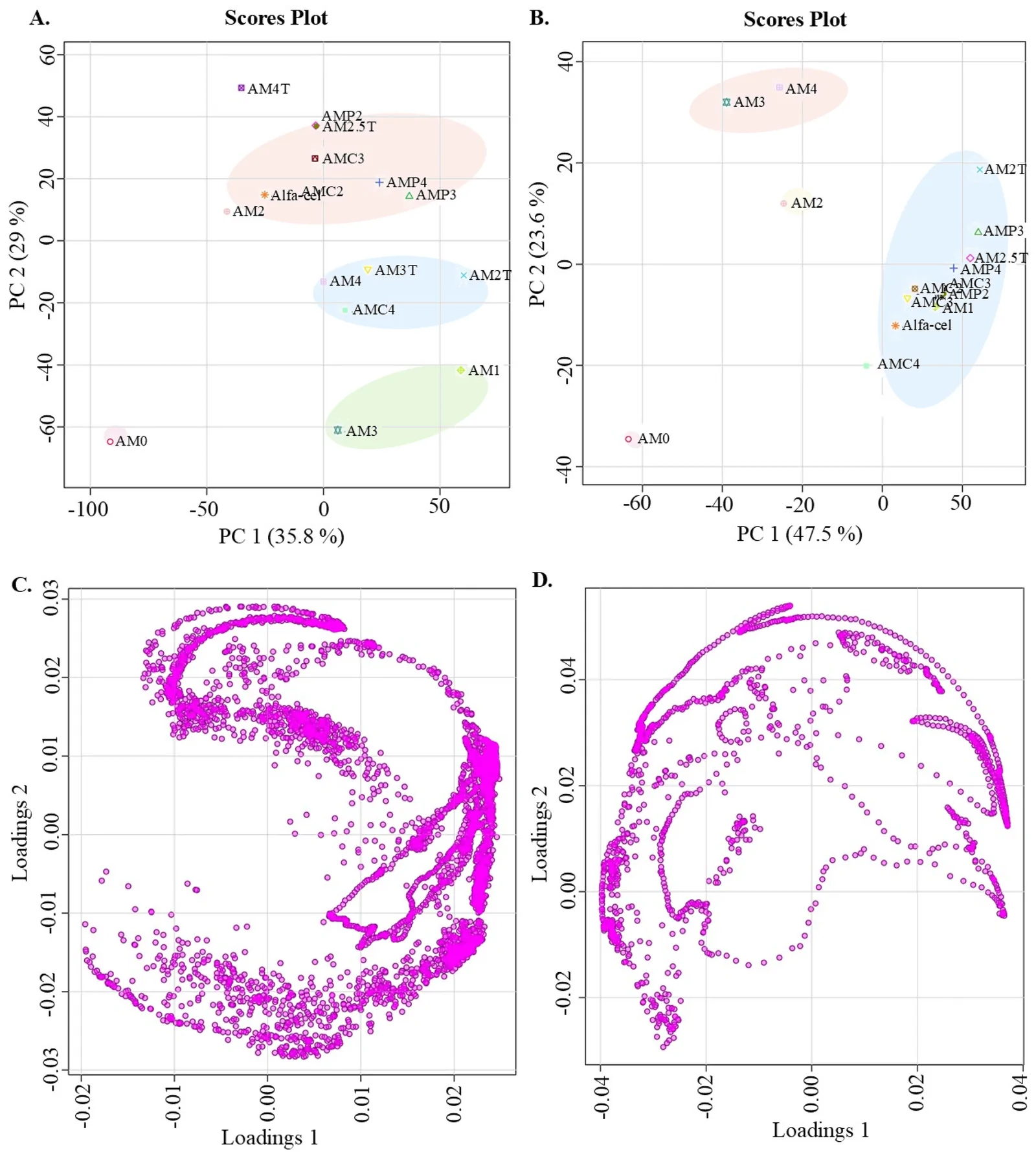
Illustration of the PCA results of FT-IR data of the cellulose samples: Scores in (**A**) in the range of 4000–600 cm^−1^ and (**B**) in the range of 1800 to 600 cm^−1^. Loading graphs: (**C**) for the PCA in the range of 4000–600 cm^−1^ and (**D**) for the PCA in the range of 1800 to 600 cm^−1^. The loadings driving the separation of AM0 are the pectin-associated bands at ~1737 cm^−1^ (ester C=O) and at 1635 cm^−1^ (carboxylate/O–H bending).

To further investigate the spectral similarities and differences among the cellulose-rich samples and to identify the spectral features responsible for their discrimination, principal component analysis (PCA) was performed on the FT-IR data. The scores plot obtained from the full spectral range (4000–600 cm^−1^) is shown in Figure 4A. The first two principal components explained 64.8% of the total variance (PC1 = 35.8% and PC2 = 29.0%), indicating that a substantial proportion of the spectral variability could be represented in the two-dimensional scores plot. The distribution of the samples revealed that the spectral differences were not randomly distributed, but rather reflected differences among the samples associated with their processing history and chemical composition.

Most of the treated samples were located in the positive region of PC1, although their positions along PC2 varied considerably. A group comprising AM2, AM2T, AMC2, AMC3, AM3P, and AMP4 was mainly located in the upper-right region of the scores plot, whereas AM3 and AM1 were shifted toward lower PC2 values. AM4, AM3T, and AMC4 occupied an intermediate region, while AM0 and AM4T were clearly separated from the main distribution. This pattern indicates that, although the samples shared the characteristic spectral features of cellulose-rich materials, subtle differences in their chemical composition and molecular organization remained after processing.

Importantly, the separation observed in the PCA did not follow a simple trend in the crystallinity index (CI) calculated from the 1425/896 cm^−1^ intensity ratio. Samples with similar CI values were not necessarily located close to each other in the scores plot, whereas samples occupying different regions could exhibit comparable CI values. This indicates that the overall FT-IR spectral variability captured by PCA cannot be attributed solely to changes in the crystallinity-related bands. Instead, the PCA incorporates the contribution of the entire spectrum, including bands associated with cellulose conformation, hydrogen bonding, glycosidic linkages, residual non-cellulosic components, and adsorbed water. Therefore, the PCA provides a more comprehensive description of spectral similarity than the single-band intensity ratio used to estimate CI.

The distinct position of AM0 is particularly relevant. As the untreated starting biomass, AM0 retained a considerably higher proportion of non-cellulosic components, particularly pectin, compared with the processed samples. Consequently, its spectrum differed substantially from those of the cellulose-enriched materials, resulting in its pronounced separation along both PC1 and PC2. In contrast, the relatively close distribution of most processed samples suggests that the alkaline treatments substantially reduced the spectral contribution of the components initially responsible for the differentiation of the raw biomass.

A second PCA was performed using only the fingerprint region (1800–600 cm^−1^), where many of the characteristic vibrations of polysaccharides are located (Figure 4B). In this case, PC1 and PC2 explained 47.5% and 23.6% of the variance, respectively, accounting for 71.1% of the total variability. The restriction of the analysis to the fingerprint region resulted in a more compact distribution of most treated samples, suggesting that their spectral profiles became considerably more similar after processing. AM0 remained clearly isolated from the main cluster, whereas AM3 and AM4 were separated predominantly along PC2. These results indicate that the spectral differences among the treated samples are relatively subtle when compared with the pronounced compositional difference between the untreated biomass and the cellulose-rich materials.

The loading distribution provides additional information regarding the spectral features responsible for these patterns (Figure 4D). In particular, the regions around 1737 and 1635–1620 cm^−1^ contribute strongly to the discrimination of AM0. The band near 1737 cm^−1^ is associated mainly with the C=O stretching vibration of esterified carboxyl groups in pectin, whereas the absorption around 1635–1620 cm^−1^ may include contributions from carboxylate groups and O–H bending of adsorbed water. The greater contribution of these spectral features to the position of AM0 is consistent with the higher abundance of pectic material in the untreated biomass. Their substantially reduced contribution in the processed samples supports the effectiveness of the alkaline treatment in removing or reducing pectin-associated spectral features.

The PCA based on selected spectral variables (Figure S5) further supports this interpretation. The variables included not only bands associated with pectin but also vibrations characteristic of cellulose, such as those near 896, 1100, 1158, 1200, 1336, 1364, and 1420 cm^−1^, as well as bands related to O–H and C–H stretching around 3336–3390 and 2900 cm^−1^, respectively. The marked separation of AM0 in this analysis indicates that its spectral profile was strongly influenced by the presence of non-cellulosic components. After alkaline processing, the samples became considerably more similar with respect to these selected spectral features, although some differences remained among the treatments.

Interestingly, the separation of AM3 and AM4 in Figure 4B suggests that the alkaline processing conditions can also induce more subtle changes in the cellulose-rich fraction that are not necessarily reflected by the CI alone. Such differences may arise from changes in hydrogen bonding, cellulose chain organization, accessibility of hydroxyl groups, or the relative contribution of residual non-cellulosic constituents. Thus, the PCA should not be interpreted as direct evidence of differences in crystallinity but rather as evidence that the FT-IR spectra retain compositional and structural information beyond that captured by the conventional CI calculation.

Overall, the PCA demonstrates that the major source of spectral discrimination is the difference between the untreated biomass and the processed cellulose-rich samples, particularly due to pectin-associated absorptions. Among the treated samples, the remaining separation is less pronounced and reflects subtle differences in their spectral profiles. These results also highlight that the interpretation of FT-IR-based crystallinity indices should be made cautiously, since the intensity of the bands used in such calculations can be influenced by factors other than cellulose crystallinity, including residual non-cellulosic components and changes in the local chemical environment of cellulose. Therefore, the PCA complements the CI analysis by demonstrating that the spectral differences among the samples cannot be reduced to a single crystallinity-related intensity ratio.

Concurrently, AM0 is displaced away from regions dominated by cellulose backbone and glycosidic vibrations (~1158–1100 cm^−1^ and the 896 cm^−1^ fingerprint), reflecting a relatively higher pectin contribution compared to crystalline cellulose signals. The box plot for the 1737 cm^−1^ band (Figure S3C) further illustrates this trend: AM2.5T (13) and AM4 (5) cluster near the alpha-cellulose standard (16), whereas AM4T (15) and AM1 (2) are closer to AM0 (1), consistent with residual pectin presence.

### 2.3. Solid-State ^13^C-NMR Spectroscopy

The ^13^C NMR spectra of the cellulose samples display the characteristic resonances of the glucopyranose ring. These signals appear at ~105 ppm for C1, ~79–94 ppm for C4, ~61–63 ppm for C6, and ~71–73 ppm for C2, C3, and C5 [4]. Among them, the C4 resonance is commonly used to evaluate cellulose crystallinity because it can be resolved into two components corresponding to crystalline (~88 ppm) and amorphous (~84 ppm) domains [32].

**Figure 5:**
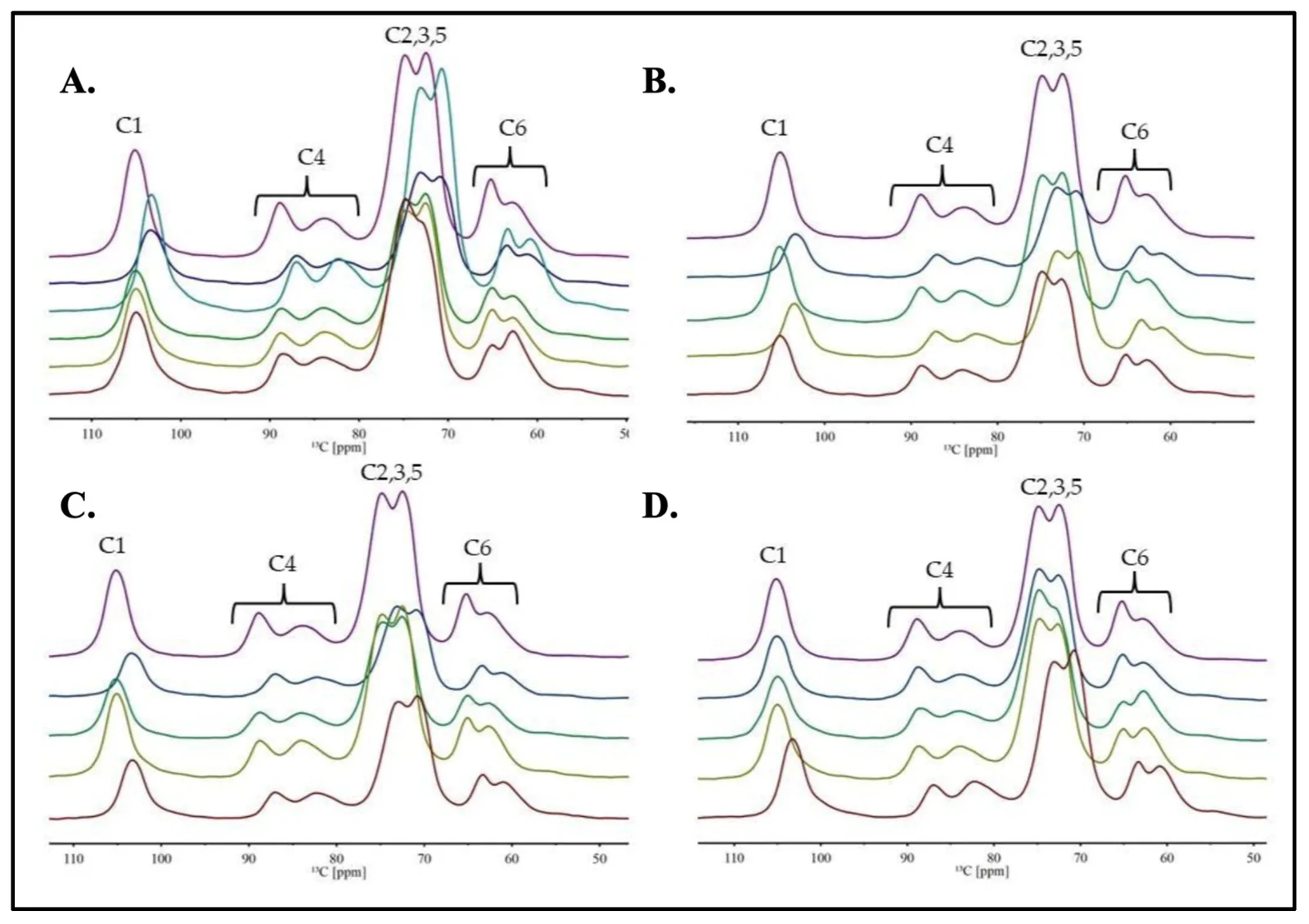
^13^C ssNMR of cellulose samples under different biomass processing. (**A**) times: AM3—red, AM2.5T—light green, AM3T—dark green, AM4T—light blue, AM2T—dark blue, and alfa-cellulose—purple; (**B**) NaOH concentrations: AM2T (NaOH 3%)—blue, AMC2 (NaOH 5%)—dark green, AMC3 (NaOH 7%)—light green, AMC4 (NaOH 9%)—red, and alfa-cellulose—purple; (**C**) S/L ratios: AMP4 (S/L ratio 1/25)—red, AMP3 (S/L ratio 1/20)—light green, AMP2 (S/L ratio 1/15)—dark green, AM2T (S/L ratio 1/10)—blue, and alfa-cellulose—purple; (**D**) Temperatures: AM1—red, AM2—light green, AM3—dark green, AM4—blue, and alfa-cellulose—purple.

Comparisons of the spectra obtained under different processing conditions are shown in Figure 5. In several samples, the C1 resonance shifted from ~105 ppm to ~103 ppm. This shift was observed for time variations in AM4T and AM2T, for NaOH concentration changes in AM2T (3% NaOH) and AMC2 (5% NaOH), for S/L ratio variations in AMP3 and AMP2, and for temperature variations in AM1. These small variations in the ^13^C CP/MAS chemical shifts were first examined considering possible referencing effects. After re-referencing the spectra using the C1 signal as an internal reference, the differences remained, particularly for the C4 and C6 carbons. These carbons are known to be highly sensitive to cellulose crystallinity and hydrogen-bonding patterns, suggesting that the observed shifts reflect structural differences rather than referencing artifacts. One possible explanation is a higher residual pectin content in these samples. Pectin presents characteristic ^13^C-NMR resonances at ~101 ppm (C1) and ~79 ppm (C4) associated with D-galacturonic acid units.

In the C4 region, the crystalline signal at ~88 ppm is more intense than the amorphous peak at ~84 ppm for samples AM3, AM4, AMP3, AMC2, AMC3, AMC4, and AM2T, as well as for the α-cellulose standard. In contrast, the remaining samples show amorphous peak intensities at ~84 ppm comparable to or slightly higher than the crystalline signal, indicating lower crystallinity. The internal chemical shift separations of the C4 and C6 resonances were also analyzed, as these parameters are widely used to assess structural order in cellulose (Table 1).

No differences were detected in the C6 splitting among the samples. In contrast, the crystalline–amorphous C4 splitting indicates that most samples retain the supramolecular organization of α-cellulose. Slight reductions in the ΔC4 values for AM1 and AM4 suggest a modest increase in local disorder, whereas AM3T, AM4T, and AMC2 exhibit more pronounced changes, particularly in the amorphous C4 region. Overall, the treatments mainly affect the less ordered domains of cellulose without inducing polymorphic transformations or complete loss of crystallinity.

**Figure 6:**
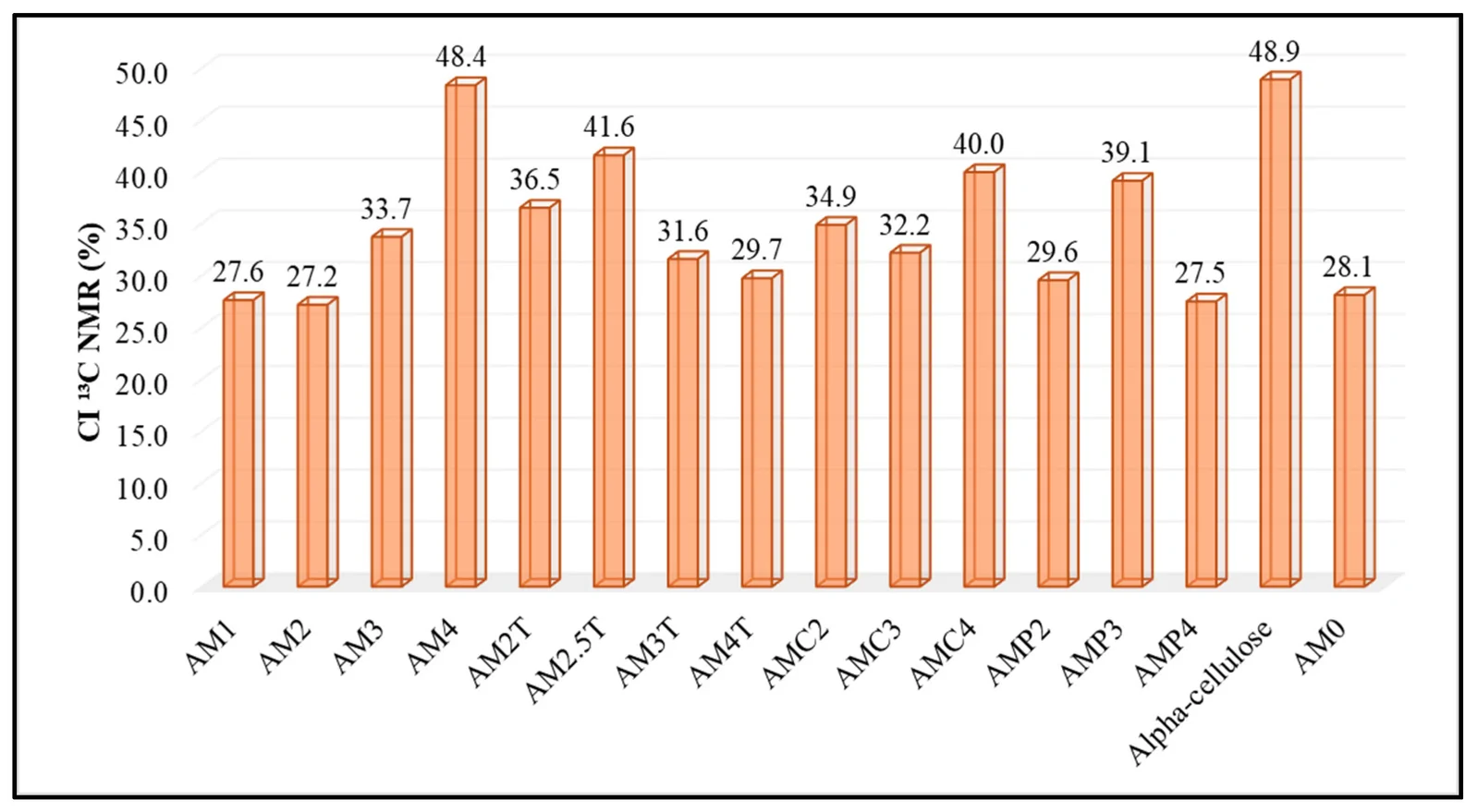
Crystallinity indices (CIs) of the obtained cellulose-based samples by using deconvolution of the ^13^C-NMR C4 peak.

The crystallinity indices (CIs) obtained are presented in Figure 6. The highest CI was 48.91% for the α-cellulose standard, followed closely by 48.35% for the AM4 sample, consistent with the intensity of the C4 peaks discussed previously. The C4 region of AM4 was the most similar to that of α-cellulose. An increase in temperature led to higher CI values, as observed for samples AM1, AM2, AM3, and AM4, which exhibited CIs of 27.63%, 27.19%, 33.73%, and 48.35%, respectively, highlighting the importance of temperature during cellulose fabrication. Higher temperatures in alkaline treatments promote the removal of hemicelluloses, lignin, pectin, and other extractives, while moderate heating can reduce amorphous regions and enrich crystalline cellulose [37]. Processing time also influenced CI, with the highest value observed at 150 min (AM2.5T). Similarly, NaOH concentration affected CI, with the highest CI achieved using 9% *w*/*w* NaOH (AMC4). These results align with Rodsamran and Sothornvit [38], who reported that alkaline concentration, temperature, and processing time affect cellulose yield, α-cellulose content, and whiteness index; our findings indicate that CI is also sensitive to these fabrication parameters.

**Figure 7:**
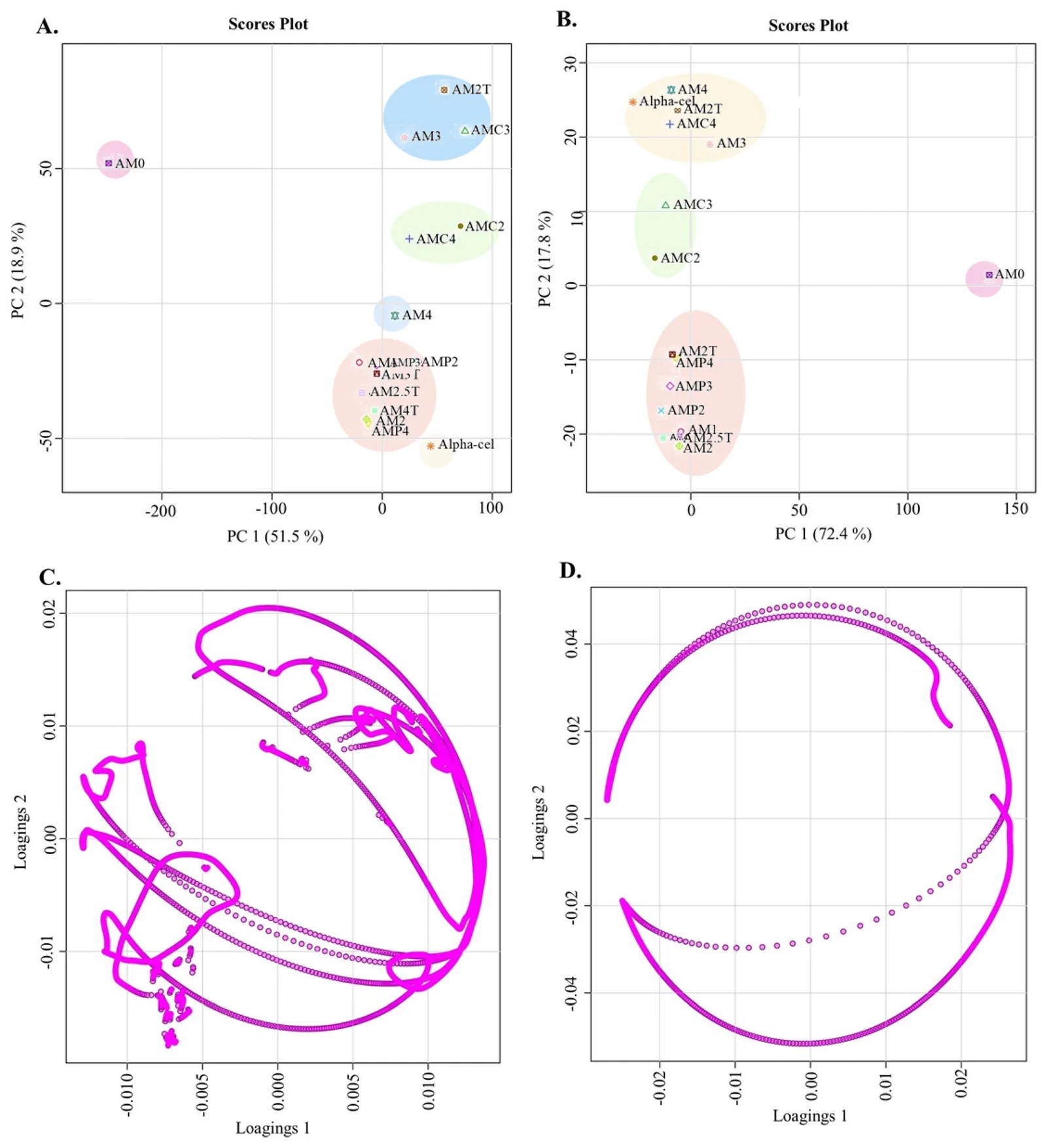
Illustration of the PCA results on the solid-state ^13^C-NMR data of the cellulose samples obtained from the two regions: Scores plots in (**A**) 40 ppm to 120 ppm and (**B**) 80 ppm to 94 ppm and the corresponding Loadings plots in (**C**) 40 ppm to 120 ppm and (**D**) 80 ppm to 94 ppm.

PCA was applied to the cellulose samples to better understand the effect of each treatment (Figure 7). Two separate analyses were performed: the first using the region of cellulose peaks (120–40 ppm, Figure 7A), and the second focusing on the C4 region (79–92 ppm, Figure 7B). In the first PCA, PC 1 and PC 2 explained approximately 70.4% of the variance. The AM0 sample was clearly isolated on the far right, indicating that it is chemically and structurally distinct from all other samples. Samples AM4, AMC4, AMC2, AMP2, AM3, AM2T, and AMC3 clustered together with α-cellulose at positive PC 1 values, while the remaining samples were grouped at negative PC 1 values, forming a tight, overlapping cluster. This distribution suggests strong similarity among these treatments, with only subtle structural differences.

In the PCA focused on the C4 region, 90.2% of the variance was explained. Again, AM0 was isolated at positive PC 1 values. Samples AM4, AM3, AMC4, AM2T, and α-cellulose clustered at the top (positive PC 2 values), with AM4 and AMC4 closest to α-cellulose, confirming their structural similarity to the standard, as also indicated by the CI results. In contrast, samples AM1, AM2, AM3T, AM4T, AMP2, AMP3, and AMP4 formed a cohesive cluster at negative PC2 values, reflecting strong chemical and structural similarity among them. AMC2 and AMC3 were positioned between these two groups, showing intermediate characteristics.

We performed a PCA using selected ^13^C-NMR variables corresponding to cellulose peaks at 81.4, 83.1, 84.2, 88.5, 88.7, and 89 ppm (Figure S4), and evaluated the intensity of these peaks across the samples (Figures S5 and S6). Overall, most samples exhibited spectral characteristics in the crystalline region that were similar to α-cellulose (class 16), particularly around ~89 ppm, indicating a predominance of the α phase. Notably, the AM4 sample (class 4) was closer to α-cellulose at 88.5 ppm and 89 ppm, further confirming its higher similarity to the standard in terms of crystalline structure.

### 2.4. XRD Analysis

XRD is frequently applied to investigate how various treatments affect cellulose structure and to correlate crystallinity variations with performance in industrial and biotechnological applications. For example, ionic liquid pretreatment can reduce CI and even induce cellulose II formation, thereby improving enzymatic hydrolysis efficiency [39]. In cellulose nanocrystal (CNC) production, XRD is essential to confirm the selective degradation of amorphous regions. Extraction from apple pomace (AP), for instance, increased CI from 45% in the raw biomass to 78% in CNCs, enhancing nanomaterial rigidity [40]. These examples highlight XRD not only as a structural characterization tool but also as a means of linking cellulose crystallinity to process efficiency in biomass valorization.

**Figure 8:**
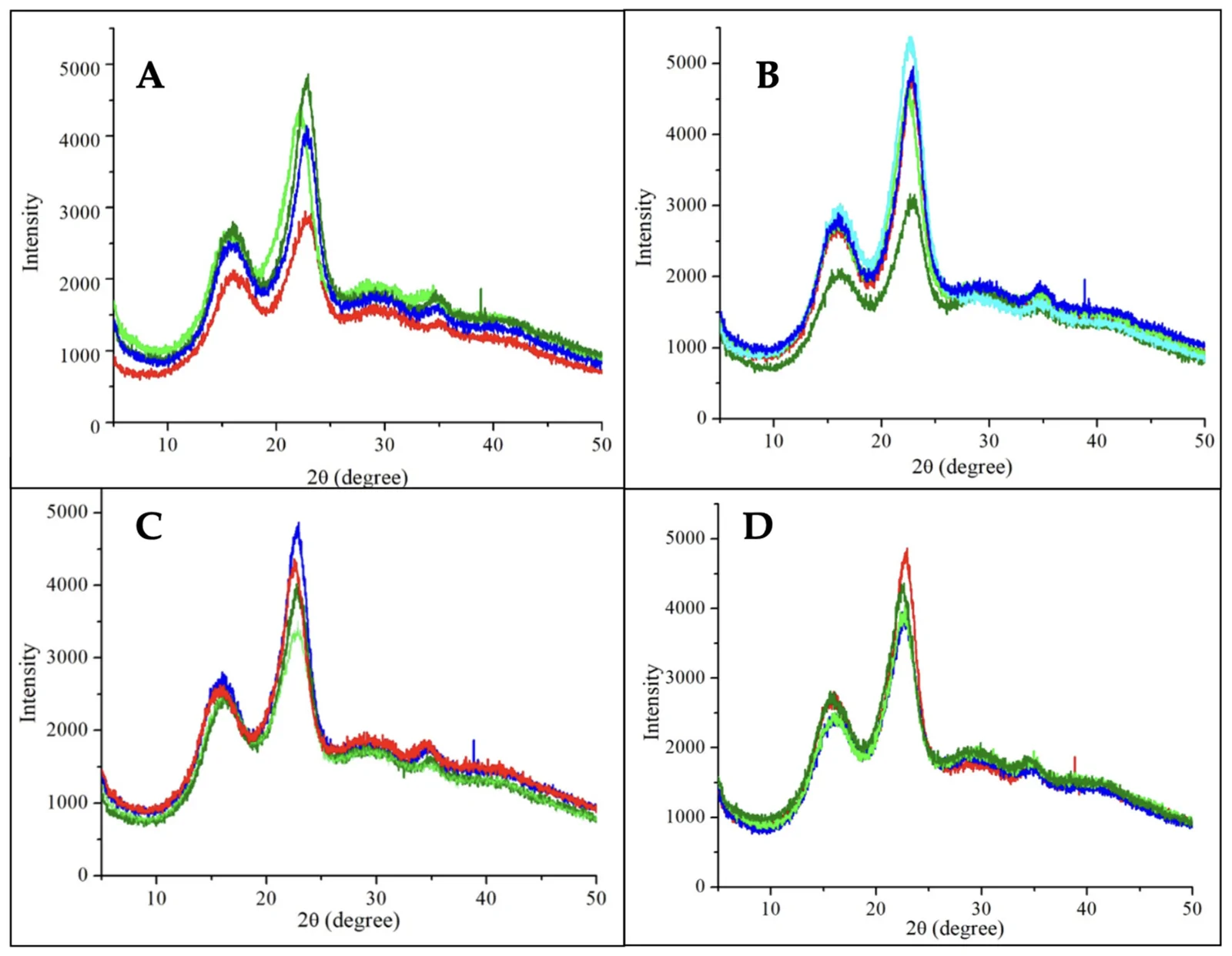
X-ray diffraction pattern of cellulose samples under different alkaline processing conditions. (**A**) temperatures: AM1—red, AM2—light green, AM3—dark green and AM4—blue; (**B**) processing times: AM3—red, AM2.5T—light green, AM3T—dark green, AM4T—light blue, AM2T—dark blue; (**C**) NaOH concentrations: AM2T (NaOH 3%)—blue, AMC2 (NaOH 5%)—dark green, AMC3 (NaOH 7%)—light green, AMC4 (NaOH 9%)—red; (**D**) S/L ratios at the range of 1800–600 cm^−1^: AM2T (1/10, red), AMP2 (1/15, blue), AMP3 (1/20, light green) and AMP4 (1/25, dark green).

The diffraction pattern of cellulose is characterized by sharp peaks corresponding to ordered crystalline domains, superimposed on a diffuse background from amorphous regions. The positions and intensities of these peaks vary depending on the cellulose allomorph: typical cellulose reflections occur around 2θ ~15.9° and ~22.6°, corresponding to the (1ī0), (110), and (200) planes [6,8,20]. The diffraction pattern of the cellulose samples are shown in Figure 8. Overall, the samples exhibited similar diffraction profiles, with the main differences being observed in the relative intensities of the characteristic reflections. These variations may be associated with changes in the relative contribution of crystalline and amorphous domains resulting from the alkaline pretreatments. 

**Figure 9:**
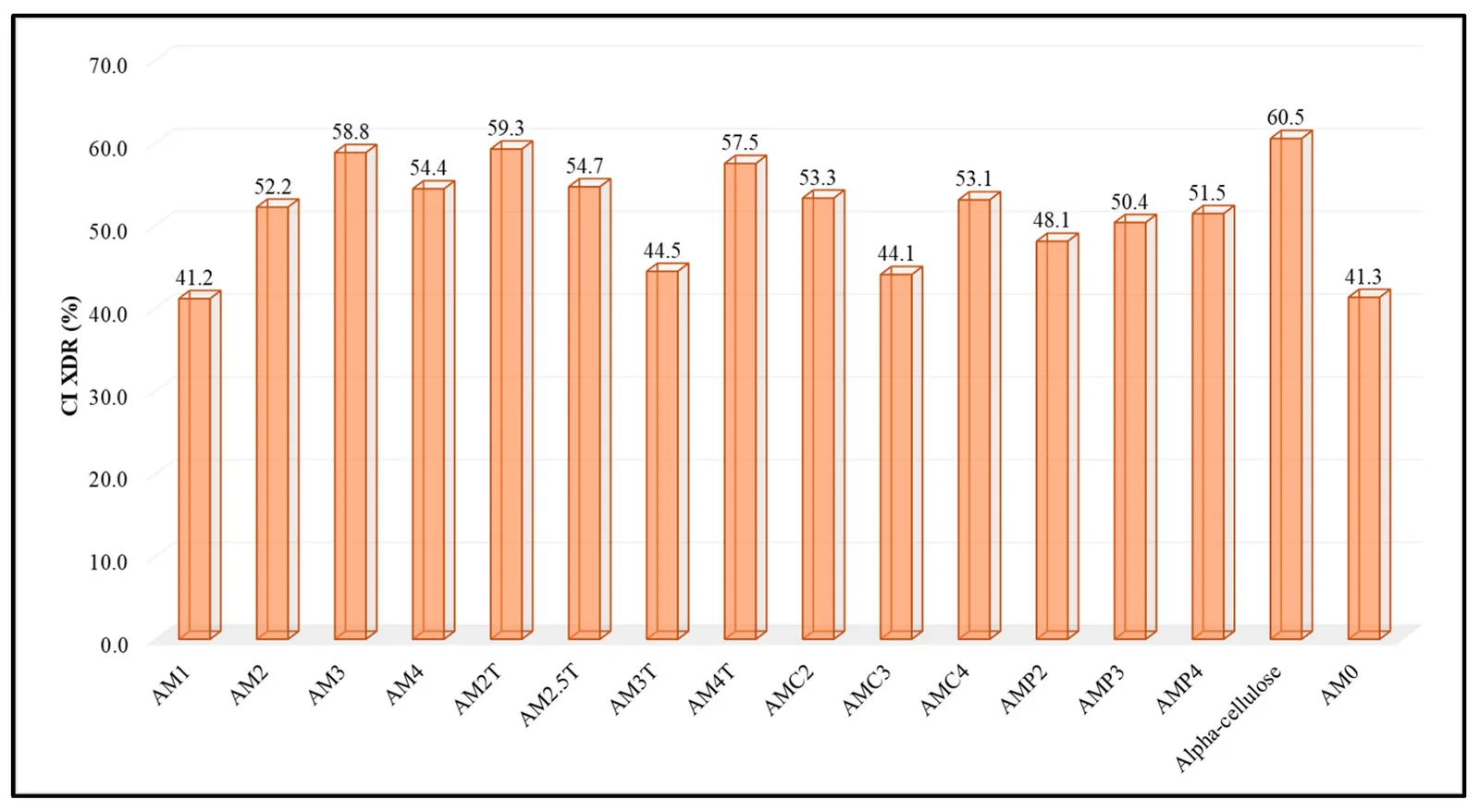
Crystallinity indices (CIs) of cellulose-based samples according to XRD and the Segal equation.

In this study, CI values were calculated using the Segal equation. It is important to note that the CI calculated from XRD using the Segal method is an empirical and semi-quantitative parameter, in which the intensity of the main crystalline reflection is considered relative to the intensity attributed to the amorphous contribution [41]. Therefore, variations in the amorphous contribution, as well as peak overlap, crystallite size, and preferential orientation, can influence the resulting CI values and should be considered when interpreting differences among samples [12,19,38]. As shown in Figure 9, these values were higher than those obtained from ^13^C-NMR but lower than those from FT-IR. Limitations of the Segal method arise mainly from the overlap of amorphous contributions with crystalline reflections, which can lead to CI overestimation [16,22]. The presence of different cellulose polymorphs also affects interpretation: in cellulose I, the (200) peak is well resolved, whereas in cellulose II, structural rearrangement during mercerization or regeneration shifts the main reflection and alters peak intensities, making direct CI comparisons across allomorphs less reliable [8].

**Figure 10:**
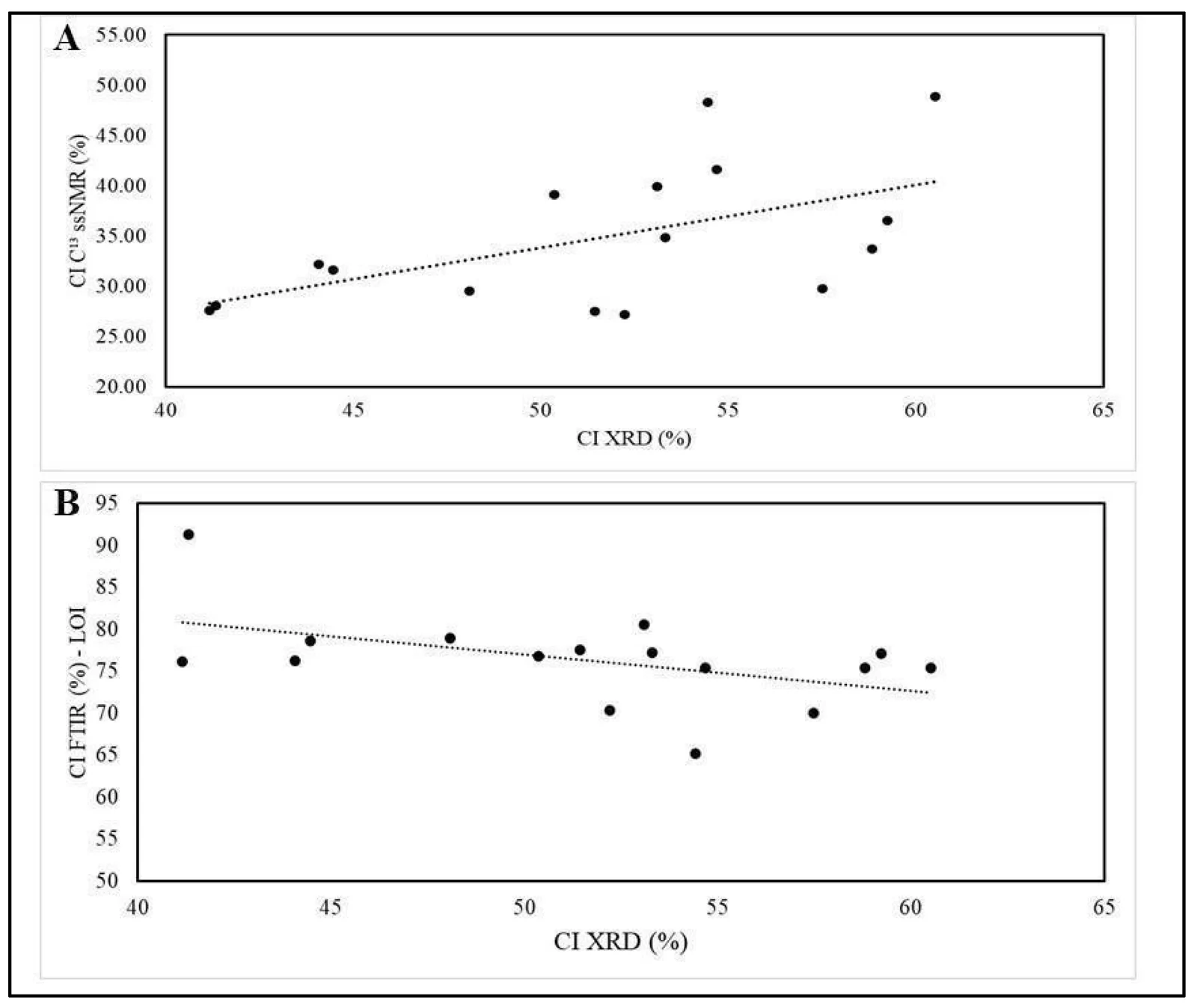
Correlation graph between CI calculated with ^13^C ssNMR—C4 peak deconvolution and XRD—Segal method (**A**) and with FT-IR—LOI and XRD—Segal method (**B**).

The crystallinity index (CI) values obtained from XRD were compared with those calculated from ^13^C-NMR and FT-IR to evaluate consistency across techniques. Pearson’s correlation coefficient (r) was calculated, yielding a value of 0.5 for the XRD vs. ^13^C-NMR comparison, indicating a moderate positive correlation (Figure 10A). This agreement is evident in the lower CI values observed for samples processed at lower temperatures (AM1 and AM2), which were consistently reflected by both techniques.

However, discrepancies were observed for samples where the processing time was varied. For instance, ^13^C-NMR indicated the highest CI for AM2.5T, whereas XRD suggested AM2T had the highest CI. Notably, the AM4T sample exhibited a large difference between techniques: a very low CI according to ^13^C-NMR but a high CI according to XRD, suggesting that the Segal method may overestimate CI in some cases.

The correlation between XRD and FT-IR-derived CI was negative (r = −0.5) (Figure 10B), indicating that higher LOI values (%) from FT-IR often correspond to lower CI values from XRD. For example, AM0 and AM1 showed LOI values of 91.3% and 76.1% (FT-IR), respectively, whereas the corresponding XRD CI values were 41.3% and 41.2%. This observation highlights that LOI is more sensitive to residual non-cellulosic components, while XRD provides a more conservative measure of true cellulose crystallinity.

### 2.5. Integrated Discussion of the Characterization Results

Taken together, the characterization results indicate that the alkaline pretreatments induced structural and compositional changes in the citrus biomass, which were reflected by the complementary analytical techniques. The combination of FT-IR, ^13^C-NMR, and XRD provided complementary information on cellulose structural organization. FT-IR-derived indices, such as the lateral order index (LOI), are sensitive to changes associated with residual non-cellulosic components and molecular organization, whereas ^13^C-NMR provides information on the relative organization of crystalline and amorphous cellulose domains. XRD, in turn, provides information on the long-range ordering of the cellulose phase. Overall, the three techniques indicated a higher degree of cellulose structural organization in AM4 and AMC4, which also showed greater similarity to α-cellulose. These results indicate that the processing conditions, including temperature, treatment time, and NaOH concentration, can substantially influence the structural organization of cellulose. However, because these techniques probe different structural features and rely on distinct analytical principles, some differences in the trends observed among the samples are expected.

This complementary nature of the techniques is particularly evident for the untreated AM0 sample. Its CI obtained by XRD was not higher than that of the other samples, despite the higher LOI observed by FT-IR. This discrepancy may indicate that the FT-IR-derived LOI is more sensitive to the presence of residual non-cellulosic components in the untreated biomass than the XRD-based CI. In addition, the XRD-based CI should be interpreted with caution because variations in the contribution of amorphous components to the diffraction profile may influence the calculated values, particularly when samples differ in their relative proportions of cellulose and non-cellulosic constituents. Nevertheless, FT-IR-derived indices should be interpreted as indirect or semi-quantitative indicators of cellulose structural organization rather than as direct equivalents of the crystallinity index obtained by X-ray diffraction. Indeed, values derived from FT-IR spectral ratios may be affected by spectral acquisition conditions, baseline correction, normalization procedures, sample preparation, and the chemical composition of the analyzed material. Moreover, different FT-IR crystallinity indices probe distinct aspects of cellulose organization and may not necessarily change in the same direction following a given treatment. Therefore, FT-IR and XRD should be considered complementary techniques: FT-IR is particularly useful for monitoring changes in cellulose-associated functional groups, molecular order, and hydrogen-bonding interactions, whereas XRD provides information on the relative crystalline and amorphous organization of the cellulose phase [17].

## 3. Materials and Methods

### 3.1. Materials

Commercial α-cellulose was obtained from Sigma-Aldrich (St. Louis, MO, USA). All other reagents, including sodium hydroxide (NaOH), hydrochloric acid (HCl), and hydrogen peroxide (H_2_O_2_), were of analytical grade and purchased from Dinamica (São Paulo, Brazil). Deionized water was used in all experiments. The starting material, depectinated orange biomass (coded as AM0), was supplied by a pectin factory located in Limeira, São Paulo, Brazil.

### 3.2. Biomass Characterization

The contents of neutral detergent fiber (NDF), acid detergent fiber (ADF), and acid detergent lignin (ADL) were determined following the procedures recommended by ANKOM Technology (New York, NY, USA), using the filter bag technique (A200 and A200I) [42]. Briefly, the samples were weighed into filter bags and subjected sequentially to the appropriate detergent treatments for the determination of NDF and ADF. For lignin determination, the residue obtained after the ADF procedure was treated with 72% sulfuric acid, following the standard ADL procedure. After each extraction step, the filter bags containing the insoluble residues were washed, dried to constant mass, and weighed according to the manufacturer’s instructions. The NDF, ADF, and ADL contents were calculated based on the mass of the respective residues and expressed as percentages of the sample dry matter, following the equation: [weight of dried bag with fiber after extraction − (W1 × C1)]/W2 × 100, where W1 is the bag tare weight, C1 is the blank bag correction (ratio between average of final oven-dried weight and weight of the original blank bag) and W2 is the sample weight.

The cellulose content was calculated from the difference between ADF and ADL, according to: Cellulose (%) = ADF (%) − ADL (%); Hemicellulose content was calculated from the difference between NDF and ADF: Hemicellulose (%) = NDF (%) − ADF (%). Lignin content was determined from the ADL fraction obtained after the acid detergent procedure. The values were expressed as a percentage of the dry matter of the samples.

Moisture content of the dried samples was determined according to the standard procedure of the U.S. National Renewable Energy Laboratory (NREL) [43], while ash content was determined according to AOAC methods.

Residual pectin was quantified using a colorimetric assay based on the reaction of m-hydroxybiphenyl with D-galacturonic acid (D-GalA), a monosaccharide released from pectin upon hydrolysis, producing a pink chromophore [44]. For this assay, samples were washed three times with hot distilled water, dried, and weighed (5 mg). Subsequently, 2 mL of cold concentrated sulfuric acid (H_2_SO_4_, 98%) was added, and the mixture was maintained in an ice bath under agitation. Then, 0.5 mL of distilled water was added and the mixture was allowed to stand for 5 min, followed by the addition of another 0.5 mL of water. The final volume was adjusted to 10 mL with distilled water.

An aliquot of 0.6 mL of this solution was transferred to a test tube maintained in an ice bath, and 3.6 mL of cold sodium tetraborate solution (Na_2_B_4_O_7_·10H_2_O, 0.0125 mol L^−1^ in 98% H_2_SO_4_) was added. The mixture was homogenized, heated in a boiling water bath (100 °C) for 5 min, and then cooled to room temperature (25 °C). Finally, 60 μL of a cold m-hydroxybiphenyl solution (0.15% in 0.5% NaOH) was added and the mixture was homogenized. Absorbance was measured at 520 nm using a UV–vis spectrophotometer (FEMTO—Cirrus 80, São Paulo, Brazil). A calibration curve was prepared using commercial D-galacturonic acid at concentrations ranging from 125 to 1000 μg mL^−1^.

### 3.3. Biomass Processing

The depectinated orange biomass (AM0) was initially pre-treated with 0.1 mol L^−1^ HCl at 80 °C for 60 min using a solid-to-liquid ratio of 1:10 (*m*/*v*, based on wet matter). After treatment, the residue was filtered and washed several times with hot water until neutral pH was reached.

The resulting biomass was subsequently subjected to alkaline processing under four independent experimental series designed to investigate the effects of NaOH concentration, temperature, processing time, and solid-to-liquid ratio (Table S1). For the temperature series, 3% (*m*/*v*) NaOH, a processing time of 180 min, and a solid-to-liquid ratio of 1:10 (*m*/*v*) were used, while the temperature varied from 60 to 90 °C. For the NaOH concentration series, concentrations of 3, 5, 7, and 9% (*m*/*v*) were evaluated at 80 °C for 150 min using a solid-to-liquid ratio of 1:10 (*m*/*v*). For the processing-time series, the treatment time was varied from 60 to 150 min at 80 °C using 3% (*m*/*v*) NaOH and a solid-to-liquid ratio of 1:10 (*m*/*v*). Finally, the effect of the solid-to-liquid ratio was evaluated using ratios of 1:10, 1:15, 1:20, and 1:25 (*m*/*v*), while maintaining 3% (*m*/*v*) NaOH, 80 °C, and 150 min.

The selected experimental ranges were based on conditions reported for alkaline processing of lignocellulosic biomasses and were intended to investigate the effects of processing severity on the resulting materials [25,27]. No formal statistical optimization or response-surface methodology was performed. After alkaline processing, the resulting solid fractions were bleached with 5% H_2_O_2_ at 80 °C for 60 min. The bleached materials were thoroughly washed with hot water to remove residual hydrogen peroxide and subsequently dried in a hot-air oven at 60 °C for 24 h.

### 3.4. Fourier Transform Infrared Spectroscopy (FT-IR)

FT-IR spectra of the cellulose samples were recorded using an attenuated total reflectance (ATR) accessory (CARY-630, Agilent Technologies, Santa Clara, CA, USA) over the wavenumber range of 4000–600 cm^−1^, with a resolution of 4 cm^−1^ and accumulation of 128 scans per sample. Spectra were baseline-corrected and normalized using OriginPro 8 software before further analysis.

The crystallinity index (CI) of the cellulose-based samples was estimated using the bands at ~1424 cm^−1^ (crystalline) and 896 cm^−1^ (amorphous), corresponding to the Lateral Order Index (LOI) as described in the introduction. The integrated intensity under these bands was calculated and used to report CI, according to Equation (1) [22,45].

CI (%) = 100 × A_1424_/A_896_ (1)

### 3.5. Solid-State ^13^C-NMR Spectroscopy (CP/MAS)

^13^C-NMR spectra were acquired on a Bruker Avance 400 MHz spectrometer (Bruker BioSpin, Karlsruhe, Germany) operating at 9.4 T (100.62 MHz for ^13^C). Cross-polarization magic-angle spinning (CP/MAS) experiments employed a 90° pulse of 1.5 ms, an 800 ms contact pulse, and a MAS rate of 10 kHz. Spectra were collected with 2000 scans and 3 s relaxation delays between scans. Spectra were aligned using the C1 peak at 105 ppm before analysis.

The deconvolution method of ^13^C-NMR spectra was employed to assess the crystallinity index (CI) of cellulose samples, following the procedure reported by Bernardinelli et al. [16]. This approach focuses on the C4 carbon region, with peaks between 80–86 ppm and 86–94 ppm corresponding to amorphous and crystalline cellulose, respectively (Figure 5). First, the C1 peak at 105 ppm was used as a reference. The C4 region was then deconvoluted to quantify the cellulose phases. For disordered cellulose, Gaussian peaks were fitted at 83.1 ppm and 84.2 ppm, representing accessible fibril surfaces, and at 81.4 ppm, representing inaccessible fibril surfaces. In the ordered cellulose region, a Gaussian peak at 88.5 ppm was assigned to paracrystalline cellulose, while three Lorentzian peaks represented crystalline cellulose: 87.9 ppm for the β phase, 88.7 ppm for the α + β phase, and 89 ppm for the α phase. The CI was calculated as the ratio of the integrated intensities of peaks assigned to ordered cellulose to the total integrated area of all peaks in the C4 region (ordered + disordered cellulose) [16,22].

### 3.6. X-Ray Diffraction (XRD)

XRD patterns were recorded using a Bruker D2 Phaser diffractometer (Bruker AXS SE, Karlsruhe, Germany) with Cu Kα radiation at 30 kV and 10 mA. Samples were scanned continuously over 2θ = 5–60° at a rate of 0.024° min^−1^. The crystallinity index (CI) was calculated according to the Segal method:

CI (%) = 100 × (I_002_ − I_am_)/I_002_ (2)

where I_002_ is the maximum intensity of the (002) crystalline peak at 2θ~22.7°, and Iam is the intensity of the amorphous background, typically measured at 2θ~18°.

### 3.7. Chemometric Analysis (PCA)

Principal Component Analysis (PCA) was performed using MetaboAnalyst 6.0. FT-IR spectra were pre-processed by baseline correction, normalization, log transformation, and auto-scaling before PCA. Similarly, ^13^C-NMR solid-state spectra were aligned at the C1 peak (105 ppm), and the resulting data were pre-processed using median normalization, log transformation, and auto-scaling. Each PCA was employed to explore relationships between cellulose samples and to evaluate the impact of processing conditions on crystallinity and structural features.

## 4. Conclusions

This study demonstrates that the processing conditions applied to depectinated citrus biomass systematically affect the structural organization and crystallinity of the resulting cellulose. By evaluating fourteen controlled alkaline conditions, we showed that changes in temperature, time, and applied NaOH concentration can be effectively monitored through a combination of FT-IR, solid-state ^13^C-NMR, and XRD, supported by chemometrics. A major contribution of this work is the demonstration that FT-IR and solid-state ^13^C-NMR provide complementary information on cellulose structural organization rather than interchangeable measurements of crystallinity. FT-IR offers a rapid and accessible approach for screening structural changes, although its crystallinity-related indices can be influenced by residual non-cellulosic components and experimental conditions. Specifically, the lateral order index (LOI) is determined by the intensity ratio of the bands at ~1424 and 894 cm^−1^. This index can be reported either as a dimensionless ratio or converted into a percentage (by multiplying by 100). Thus, the mathematical interpretation is equivalent in both forms; however, the literature may refer to this parameter using different terminologies, including the LOI or FT-IR-derived crystallinity index. In the context of the present study, lower LOI values indicate a lower relative contribution of the 1424 cm^−1^ band and were associated with more effective removal of non-cellulosic components during alkaline processing. Therefore, the LOI should be interpreted in conjunction with the chemical composition and other structural characterization techniques rather than as a direct equivalent of the crystallinity index obtained by XRD. The chemometrics further demonstrated the potential of combining multivariate analysis with spectroscopic data to discriminate samples obtained under different conditions and to identify spectral features associated with structural changes. The differences observed among the analytical techniques further emphasize that FT-IR, ^13^C-NMR, and XRD should not be regarded as interchangeable approaches for quantifying cellulose crystallinity. Instead, their integration provides a more comprehensive framework for evaluating cellulose structural organization in heterogeneous biomass. Our findings contribute to a better understanding of how alkaline conditions affect cellulose structure in depectinated citrus biomass and provide a practical analytical framework for screening and optimizing processes. This integrated approach may support the valorization of citrus processing residues as a source of cellulose with tailored structural properties for subsequent applications in biopolymers, composites, and other cellulose-based materials.

## Figures and Tables

**Table 1:** Internal chemical shift separations of the C4 (ppm) in ^13^C-NMR CP/MAS.

Sample	C4 Amorph (ppm)	C4 Crystalline (ppm)	ΔC-4 (ppm)	Disorder Degree
α-cellulose	83.54	88.90	5.36	-
AM2	83.85	88.77	4.92	Very low
AM3	83.88	88.89	5.01	Very low
AMC4	83.98	88.87	4.89	Very low
AM2.5T	83.74	88.78	5.04	Very low
AMP2	83.98	88.82	4.84	Very low
AMP3	83.88	88.75	4.87	Very low
AM1	82.35	87.02	4.67	Light
AM4	84.08	88.76	4.68	Light
AM3T	84.27	88.77	4.50	Moderate
AMC2	82.10	88.61	6.51	High
AM4T	82.26	88.75	6.49	High

## Data Availability

The raw data supporting the conclusions of this article will be made available by the authors on request.

## Supplementary Materials

The following supporting information can be downloaded at https://www.mdpi.com/article/10.3390/molecules31213833/s1: Table S1. Sample codes and corresponding alkaline-processing conditions. AM2T was used as the baseline condition for the NaOH concentration, processing time, and solid-to-liquid ratio series. Figure S1. Illustration of the PCA results of the cellulose-derived samples: A. Scores show the AM0 sample separated from the main cluster, and B. Corresponding loadings indicate that this separation is driven by pectin-associated bands (ester C=O at ~1737 cm^−1^ and carboxylate/O–H bending at ~1620–1635 cm^−1^). Glycosidic and cellulose backbone vibrations (~1158–1100 cm^−1^, 895 cm^−1^) showed negative loadings in PC 2. Figure S2. Box Plot of the FTIR bands related to cellulose. A. 664 cm^−1^; B. 896 cm^−1^, and C. 1100 cm^−1^. Figure S3. Box Plot of the FTIR bands related to pectin. A. 1427 cm^−1^; B. 1635 cm^−1^, and C. 1737 cm^−1^. Figure S4. A. Scores plot of the PCA of solid-state ^13^C-NMR of the cellulose samples with the peaks between 81.4 and 89.0 ppm. B. Loadings plot. Figure S5. Box Plot of the ^13^C-NMR peaks in the amorphous region of C4. A. 81.40 ppm; B. 83.10 ppm, and C. 84.21 ppm. Figure S6. Box Plot of the ^13^C-NMR peaks in the crystalline region of C4. A. 88.50 ppm; B. 87.90 ppm, and C. 89.02 ppm.
