# Supplementary material for: Cellulose Crystallinity in Orange Biomass-Derived Cellulose: Comparative Assessment by FT-IR, Solid-State 13C NMR, and X-Ray Diffraction
Source: Molecules. 2026 Oct 23;31(21):3833. doi: 10.3390/molecules31213833 (PMC13765223; doi:10.3390/molecules31213833)
Supplement: Supplementary file 1 [file molecules-31-03833-s001.zip › molecules-4523167-supplementary.pdf]

Cellulose Crystallinity in Orange Biomass-Derived Cellulose: Comparative Assessment by FT-IR, Solid-State ^{13}C NMR, and X-Ray Diffraction

Symone Costa de Castro [1,2], Matheus Signorelli Corrêa [1] and Ljubica Tasic [1,*]

[1] Laboratory of Biological Chemistry (LQB), Institute of Chemistry, Universidade Estadual de Campinas (UNICAMP), Campinas 13083-970, SP, Brazil; s232970@dac.unicamp.br (S.C.C.); m236586@dac.unicamp.br (M.S.C.); ljubica@unicamp.br (L.T.)

[2] Instituto Federal de Educação, Ciência e Tecnologia Farroupilha (IFFar), Alegrete 97555-000, RS, Brazil; symone.castro@iffarroupilha.edu.br (S.C.C.)

* Correspondence: ljubica@unicamp.br; Tel.: +551-19-3521-1106

Supplementary Information

Contens:

Table S1. Sample codes and corresponding alkaline-processing conditions. AM2T was used as the baseline condition for the NaOH concentration, processing time, and solid-to-liquid ratio series S2

Figure S1. Illustration of the PCA results of the cellulose-derived samples: A. Scores show the AM0 sample separated from the main cluster, and B. Corresponding loadings indicate that this separation is driven by pectin-associated bands (ester C=O at ~1737 cm^{-1} and carboxylate/O–H bending at ~1620–1635 cm^{-1}). Glycosidic and cellulose backbone vibrations (~1158–1100 cm^{-1}, 895 cm^{-1}) showed negative loadings in PC 2. .... S3

Figure S2. Box Plot of the FTIR bands related to cellulose. A. 664 cm^{-1}; B. 896 cm^{-1}, and C. 1100 cm^{-1}. S3

Figure S3. Box Plot of the FTIR bands related to pectin. A. 1427 cm^{-1}; B. 1635 cm^{-1}, and C. 1737 cm^{-1}. S4

Figure S4. A. Scores plot of the PCA of solid-state ^{13}C-NMR of the cellulose samples with the peaks between 81.4 and 89.0 ppm. B. Loadings plot. S4

Figure S5. Box Plot of the ^{13}C-NMR peaks in the amorphous region of C4. A. 81.40 ppm; B. 83.10 ppm, and C. 84.21 ppm. S5

Figure S6. Box Plot of the ^{13}C-NMR peaks in the crystalline region of C4. A. 88.50 ppm; B. 87.90 ppm, and C. 89.02 ppm. S5

Table S1. Sample codes and corresponding alkaline-processing conditions. AM2T was used as the baseline condition for the NaOH concentration, processing time, and solid-to-liquid ratio series.

Series	Sample	NaOH (%)	Time (min)	Temperature (°C)	S/L ratio
Temperature	AM1	3	180	60	1:10
	AM2	3	180	70	1:10
	AM3	3	180	80	1:10
	AM4	3	180	90	1:10
NaOH concentration	AM2T	3	150	80	1:10
	AMC2	5	150	80	1:10
	AMC3	7	150	80	1:10
	AMC4	9	150	80	1:10
Processing time	AM4T	3	60	80	1:10
	AM3T	3	90	80	1:10
	AM2.5T	3	120	80	1:10
	AM2T	3	150	80	1:10
Solid/liquid ratio	AM2T	3	150	80	1:10
	AMP2	3	150	80	1:15
	AMP3	3	150	80	1:20
	AMP4	3	150	80	1:25

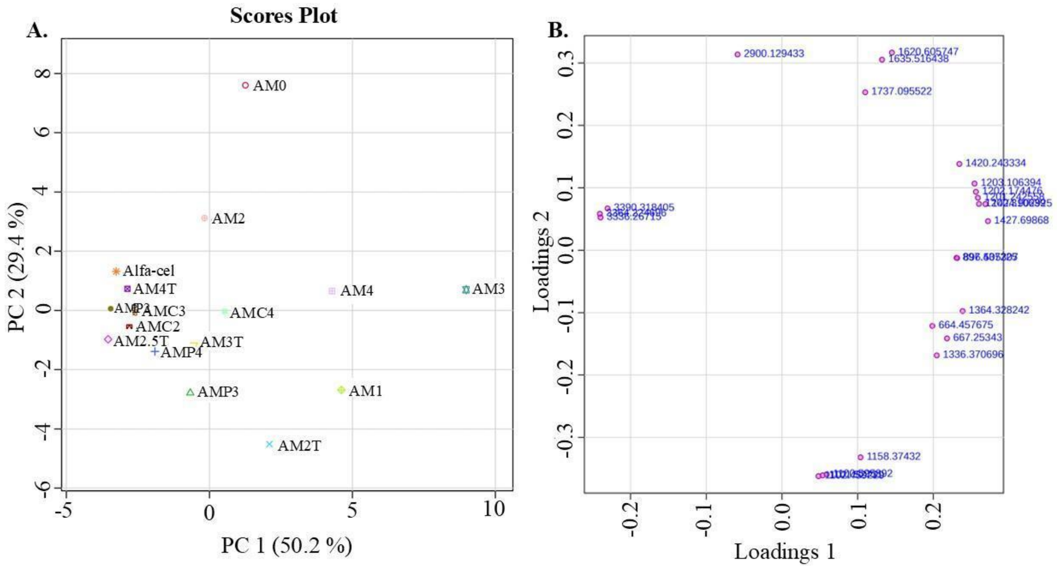

Figure S1. Illustration of the PCA results of the cellulose-derived samples: A. Scores show the AM0 sample separated from the main cluster, and B. Corresponding loadings indicate that this separation is driven by pectin-associated bands (ester C=O at ~1737 cm⁻¹ and carboxylate/O–H bending at ~1620–1635 cm⁻¹). Glycosidic and cellulose backbone vibrations (~1158–1100 cm⁻¹, 895 cm⁻¹) showed negative loadings in PC 2.

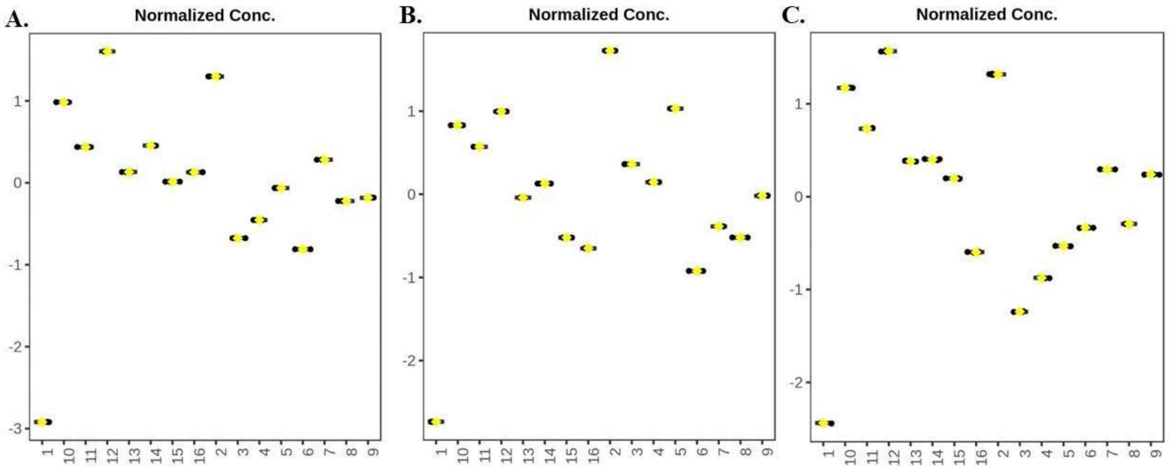

Figure S2. Box Plot of the FTIR bands related to cellulose. A. 664 cm^{-1}; B. 896 cm^{-1}, and C. 1100 cm^{-1}.

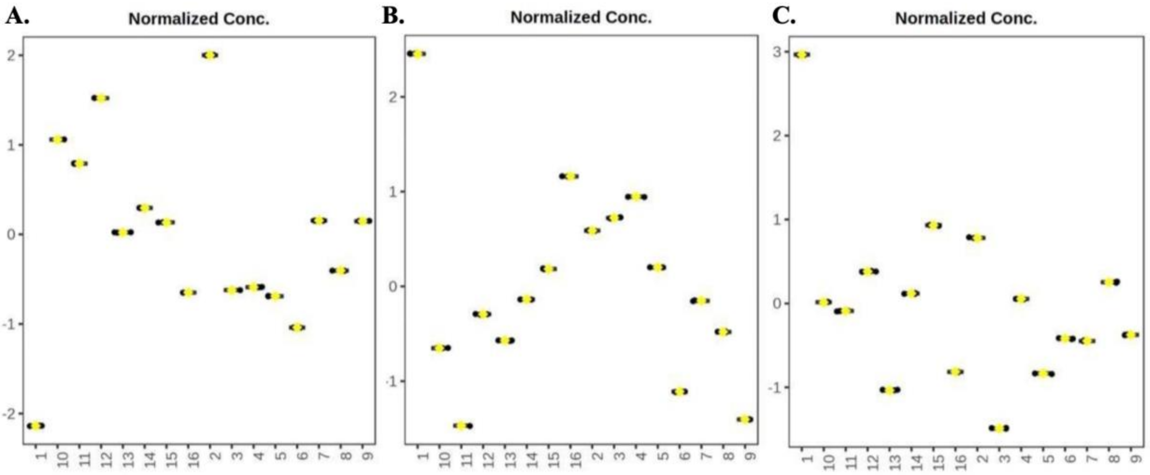

Figure S3. Box Plot of the FTIR bands related to pectin. A. 1427 cm^{-1}; B. 1635 cm^{-1}, and C. 1737 cm^{-1}.

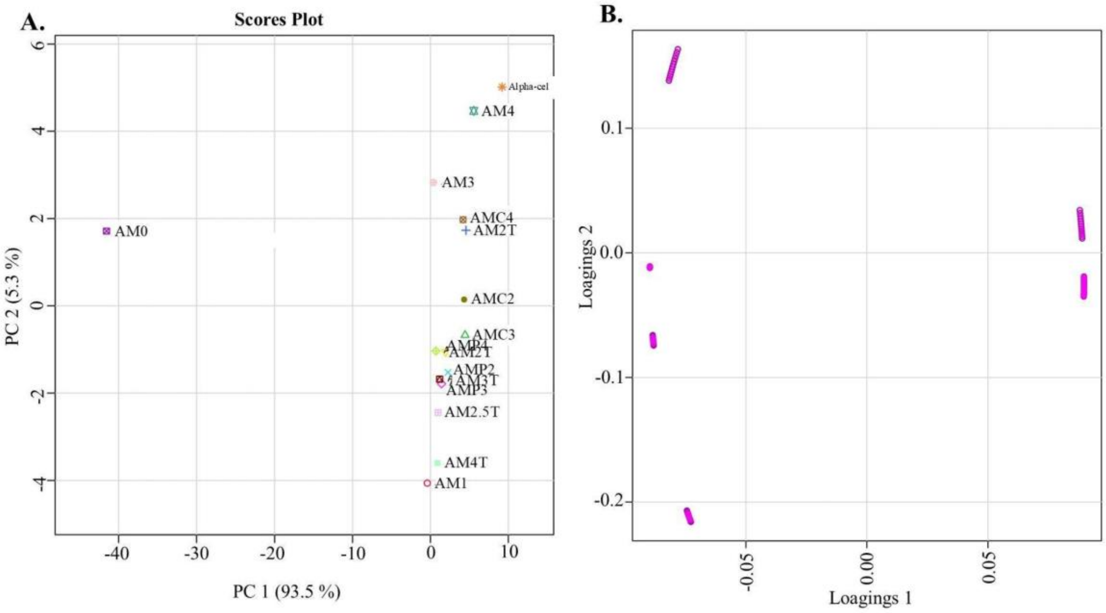

Figure S4. A. Scores plot of the PCA of solid-state ^{13}C-NMR of the cellulose samples with the peaks between 81.4 and 89.0 ppm. B. Loadings plot.

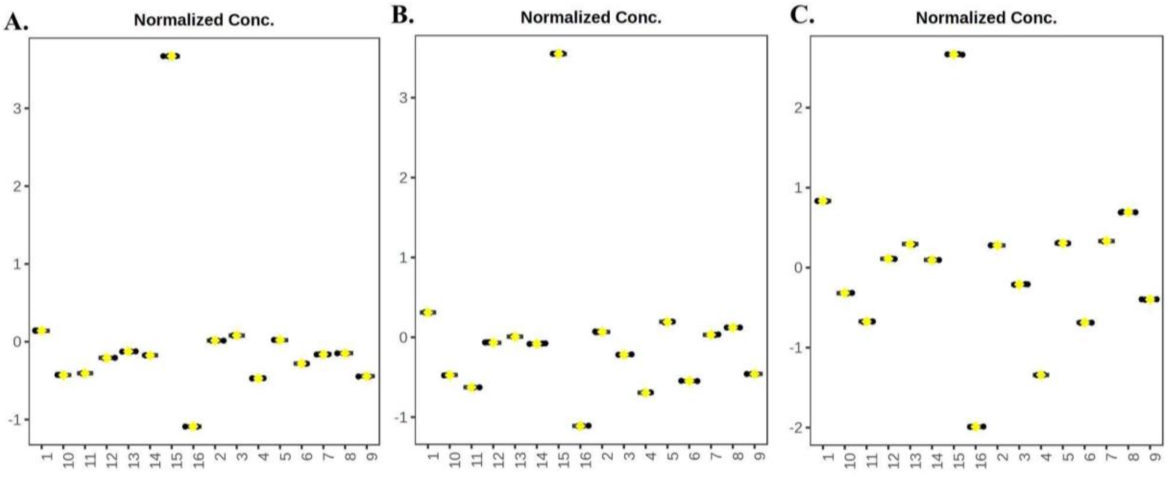

Figure S5. Box Plot of the ^{13}C-NMR peaks in the amorphous region of C4. A. 81.40 ppm; B. 83.10 ppm, and C. 84.21 ppm.

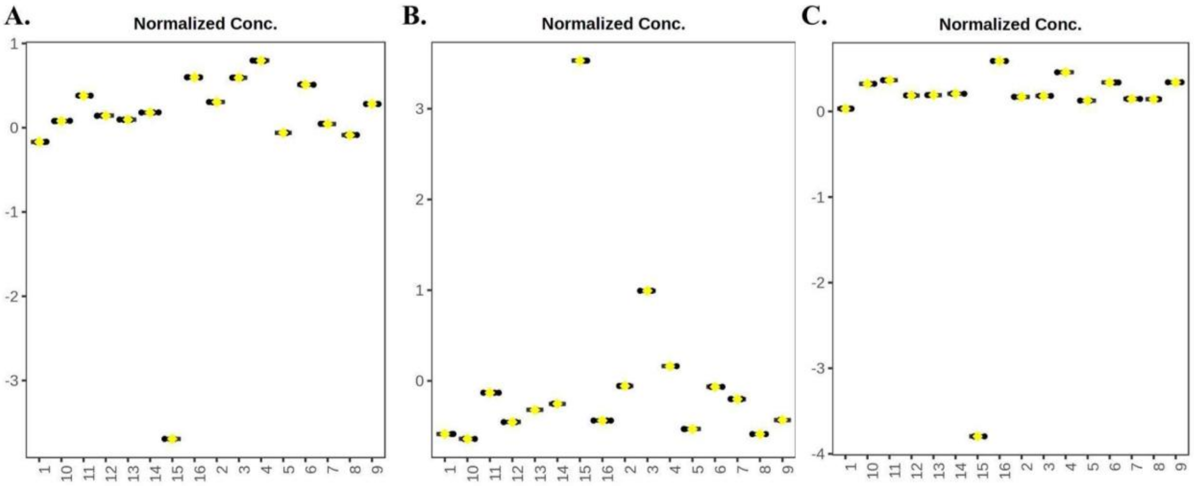

Figure S6. Box Plot of the ^{13}C-NMR peaks in the crystalline region of C4. A. 88.50 ppm; B. 87.90 ppm, and C. 89.02 ppm.
